# Discussing patients’ insurance and out-of-pocket expenses during GPs’ consultations

**DOI:** 10.1186/s12913-019-3966-8

**Published:** 2019-02-28

**Authors:** A. Victoor, J. Noordman, A. Potappel, M. Meijers, C. J. J. Kloek, J. D. de Jong

**Affiliations:** 1 Nivel (Netherlands institute for health services research), PO Box 1568, 3500 BN Utrecht, The Netherlands; 2 Radboud university medical center, Radboud Institute for Health Sciences, Department of Primary and Community Care, Nijmegen, The Netherlands; 3 Research Group Innovation of Human Movement Care, HU University of Applied Sciences, Utrecht, Utrecht, The Netherlands; 4 Maastricht University, Maastricht, The Netherlands

**Keywords:** Insurance, Out-of-pocket expenses, General practice, Decision-making, Communication, Patient involvement, Observational study, Video recording

## Abstract

**Background:**

Generally, a significant portion of healthcare spending consists of out-of-pocket (OOP) expenses. Patients indicate that, in practice, there are often some OOP expenses, incurred when they receive medical care, which are unexpected for them and should have been taken into account when deciding on a course of action. Patients are often reliant on their GP and may, therefore, expect their GP to provide them with information about the costs of treatment options, taking into consideration their individual insurance plan. This also applies to the Netherlands, where OOP expenses increased rapidly over the years. In the current study, we observed the degree to which matters around patients’ insurance and OOP expenses are discussed in the Netherlands, using video recordings of consultations between patients and GPs.

**Methods:**

Video recordings were collected from patient-GP consultations in 2015–2016. In 2015, 20 GPs and 392 patients from the eastern part of the Netherlands participated. In 2016, another eight GPs and 102 patients participated, spread throughout the Netherlands. The consultations were coded by three observers using an observation protocol. We achieved an almost perfect inter-rater agreement (Kappa = .82).

**Results:**

In total, 475 consultations were analysed. In 9.5% of all the consultations, issues concerning patients’ health insurance and OOP expenses were discussed. The reimbursement of the cost of medication was discussed most often and patients’ current insurance and co-payments least often. In some consultations, the GP brought up the subject, while in others, the patient initiated the discussion.

**Conclusions:**

While GPs may often be in the position to provide patients with information about treatment alternatives, few patients discuss the financial effects of their referral or prescription with their GP. This result complies with existing literature. Policy makers, GPs and insurers should think about how GPs and patients can be facilitated when considering the OOP expenses of treatment. There are several factors why this study, analysing video recordings of routine GP consultations in the Netherlands, is particularly relevant: Dutch GPs play a gatekeeper function; OOP expenses have increased relatively swiftly; and patients have both the right to decide on their treatment, and to choose a provider.

## Background

Healthcare can be paid for by means of a variety of financing arrangements. In some countries, healthcare costs are predominantly covered by the government; in others, people are insured against medical costs by means of a health insurance [1]. Regardless of these arrangements, rising healthcare costs mean that in several countries an increasing proportion of healthcare spending consists of out-of-pocket (OOP) expenses [1]. These expenses take three forms. Firstly, deductibles, that is how much people have to spend for health services covered by their insurance, before their insurance company pays anything. Secondly, there are co-payments, meaning payments that people make each time they use a medical service. Thirdly, there is co-insurance, meaning the percentage of costs of a covered health care service people pay after their deductible has been met. OOP expenses serve to contain collective healthcare expenditure and to increase patients’ awareness of costs by shifting some of the responsibility for healthcare spending to individual patients [2]. To avoid unexpected financial costs, patients need to take these expenses into account when they require medical care so they can evaluate the costs of alternative treatment options, which depend on their individual insurance plan [3]. However, patients indicate that they had to pay either all or part of the bill themselves when they received medical care or medication, while they thought that their insurer would reimburse all costs or that they did not adhere to prescribed health care interventions because of difficulty paying for them [4, 5] . This also applies to the Netherlands, the country of focus in the current study, where OOP expenses exist of deductibles and co-payments.

Major parts of the Dutch healthcare system (Table 1) are comparable to those in other European countries, such as the Nordic countries and the UK. However, the Netherlands is unique in that it combines elements of various healthcare systems. These elements include: 1) the gatekeeper system; 2) patients having the right to decide on their treatment and to choose a provider; 3) every Dutch citizen is required to have a health insurance; and 4) insurers may use deductibles and co-payments to steer patients to efficient providers and treatments. Because steering of enrolees gets more attention nowadays, patients become increasingly aware of the possibility to influence their OOP expenses. Additionally, the rapid increase in OOP expenses in recent years [6] in combination with a low reference point means that OOP expenses will have a relatively high impact upon people’s behaviour [7].

**Table 1:** Relevant aspects of the Dutch healthcare system

Relevant aspects of the Dutch healthcare system	
- Every Dutch citizen is required to have a basic health plan (compulsory health insurance). - Citizens can buy supplementary insurance that can reimburse the costs of additional healthcare and co-payments. - Every year enrolees are allowed to switch health plans. - A mandatory deductible of €385 per person per year in 2016–2018. - A voluntary deductible up to €500 per person per year. - Patients share some of the costs of selected services, such as medical devices, via co-payments. - Insurers are allowed to contract care providers selectively and do not fully have to reimburse those who are not contracted. - Costs of treatment might not be fully covered by an insurance plan and the level of coverage might differ per provider. - Treatments are not charged separately, but in a diagnose treatment combination (DBC). A DBC contains the whole course of treatment from people’s first consultation up to the last contact. Insurers negotiate the cost of each DBC with each individual provider. Ideally these tariffs (per provider) are made public on the insurer’s website. The publication process is still developing.	

To be able to take OOP expenses into account, patients need information about the alternative treatments available and what the implications for their own expenses are. It is generally known that most patients do not take the information provided about the quality and costs of care into account when choosing a healthcare provider or treatment [8, 9]. Instead, the general practitioner (GP) or family physician constitutes an important determinant of the course of action patients take [9, 10]. One third of the European countries use such gatekeeping to regulate access to specialist care [11]. The GP is often the first point of contact between the patient and the healthcare system and is expected to match the demands of the patients with their medical need [12]. GPs are responsible for overseeing and co-ordinating the health needs of a patient [13], providing care themselves, if possible, and authorising referral to medical specialist care if necessary [6, 13, 14]. Although an official referral is not required for primary care, which may include, as well as GPs, allied health professionals or a practice nurse working in the mental health sector, research from the Netherlands indicates that only a minority of patients visit a primary care provider, other than the GP, on their own initiative [15].

In countries with a gatekeeping system, such as the Netherlands, GPs are the most important source of information for a majority of patients [16, 17]. Because patients often rely on their GP for matters involving their health and the patient and GP often decide on the course of action that is to be taken following the consultation, GPs should provide their patients with information about treatment alternatives. Ideally, this would include the costs of the different options [12]. Although GPs are divided about if discussing costs issues with patients belongs to the profession’s job responsibilities, all providers are obliged to provide patients with the information that is relevant for them to be able to make an informed choice [18, 19]. This also applies to providers who refer patients to another provider. Because patients’ OOP expenses often depend on their type of insurance, these insurance matters should, ideally, be discussed during the consultation as well. Although patients’ involvement in decision-making has increased over time [20], literature points out that treatment costs are rarely discussed during patient-doctor consultations [21, 22].

### Research question

The research question is: To what degree do patients and GPs discuss patients’ insurance and OOP expenses during consultations in the Netherlands? Such expenses would include, for instance, whether the patient has to pay a deductible and if treatment costs are reimbursed by patients’ insurers. In the current study, we observed, in video recordings of real-life consultations between patients and their GPs, the degree to which matters around the insurance of patients and OOP expenses are discussed in the Netherlands. In the literature, different definitions of conversations relating to costs are distinguished. We use the definition “cost/coverage”, meaning discussion of the patient’s OOP expenses or insurance coverage [23]. The study gives insight into the roles of patients and GPs in discussing patients’ insurance and OOP expenses. Depending on the results, GPs and insurers could use these insights as a basis for adjusting their policy regarding the subject. GPs could plan to discuss OOP expenses more often with patients. Insurers could also investigate what information is required to allow GPs and patients to take OOP expenses into account. After all, in order to be able to channel patients to contracted providers effectively and to stimulate these to opt for efficient care, both GPs and patients need to take the costs of treatment of different providers into account.

### Scientific and social relevance

Although several papers already exist on this subject [23], most of these concern questionnaire studies [24] or focus groups [21]. They focus only on a specific disease or discipline [22, 23, 25], on medicines [26], or on a specific population group such as the elderly [27]. In the present study, we analyse video recordings of routine GP consultations involving all kinds of patients and health problems.

We focus on the situation in the Netherlands. We already mentioned that the Netherlands is unique in that it combines elements of various healthcare systems, such as the gatekeeper system and free choice of treatment and provider. Additionally, because OOP expenses were traditionally low in the Netherlands but have increased rapidly in recent years [6], OOP expenses will have a relatively high impact upon people’s behaviour [7]. For these reasons, observing real-life, GP consultations in the Netherlands adds to the existing literature and has a relevance beyond the borders of the Netherlands.

## Methods

### Recruitment of participants and procedure

Video recordings of real-life GP consultations were collected during 2015–2016 as part of a study that aimed to investigate GP-patient communication [28]. In 2015, 20 GPs (56% response) and 392 patients (77% response) from the eastern part of the Netherlands participated as part of a larger study [28]. In 2016, another eight GPs (18% response) and 102 patients (63% response) participated, spread throughout the Netherlands. These GPs were approached via the network of the researchers and through their participation in earlier studies by Nivel and the Radboud university medical center (Radboudumc). GPs and patients knew that the study was about GP-patient communication but were unaware that the discussion of patients’ insurance and OOP expenses was being analysed. Researchers from Radboudumc and Nivel visited the GPs to invite patients, collect data, and videotape consultations with an unmanned digital camera during two random days. All patients who participated filled in an informed consent form before the recording of the consultation. Prior to, and directly after, the consultation, patients completed a questionnaire about their socio-demographic characteristics and their priorities for the consultation. GPs filled in a registration form for every contact with a patient (i.e. ICPC code) and a questionnaire (e.g. their date of birth, working hours per week). In order to protect the patients’ privacy, the video data were anonymised and the video recorders were directed at the GP. Both studies adhered to Dutch privacy legislation.

### Observations

The consultations were coded by three observers (AP, MM and CK) using an observation protocol we developed to describe the degree to which patients’ insurance and OOP expenses were discussed. Part of the protocol was developed in advance based on common sense and the literature (e.g. [23]). The three observers tested the protocol together on a random sample of the videos collected. This resulted in minor changes such as adding additional subjects and/or categories when they were encountered (e.g. the practice nurse in mental health care). We assumed that when no new themes emerged the subject was covered and we considered it complete. The protocol consisted of: two questions about the mentioning of patients’ insurance and/or OOP expenses; which issues were discussed concerning insurance and/or OOP expenses, and, on whose initiative?

To assess inter-rater reliability, a random 10 % (*n* = 48) of the consultations was rated by the three observers independently. This resulted in almost perfect Kappa scores of .82 (range .77–.92) [29].

### Statistical analyses

The descriptive analyses and the inter-rater reliability calculation were performed using Stata 15. Because of the explorative and qualitative nature of the data we did not perform statistical analyses in order to determine causation.

## Results

### Background characteristics

In total, 475 consultations were recorded (recording failed of 19 consultations). The number of videotaped consultations per GP ranged from one to 29. Table 2 describes the demographic characteristics of the patients and GPs participating. The majority of both the GPs and the patients were female. GPs were, on average, 48 years old, and patients 55 years old. Of all patients, 273 (58%) received one or more referrals or prescriptions (not in table). Most of the referrals or prescriptions concerned a prescription for medication (30%). Few patients were referred to a medical professional for which no referral is needed (e.g. allied health professional, practice nurse in mental health) (4%), and no patients received a prescription for a medical aid.

**Table 2:** Background characteristics of the patients and the GPs per patient group

	Patient (*n* = 475)	GP (*n* = 28)
Age in years (M(SD))	54.8 (17.7)	47.7 (10.1)
Gender (n(%))		
Woman	273 (57.5)	16 (57.1)
Educational level (n(%))		
None^1^	11 (2.3)	0 (0.0)
Low^2^	201 (42.3)	0 (0.0)
Medium^3^	92 (19.4)	0 (0.0)
High^4^	163 (34.3)	28 (100.0)
Missing	8 (1.7)	0 (0.0)

^1^*None* no education; ^2^*Low* primary school or only vocational training; ^3^*Medium* secondary school or intermediate vocational training; ^4^*High* tertiary education

### Discussing patients’ insurance and/or OOP expenses

In 9.5% of all the consultations we observed, issues concerning patients’ health insurance and/or OOP expenses were discussed during GP consultations. Table 3 shows the issues that were discussed and how often they were discussed. The reimbursement of medication costs was discussed most often, while patients’ current health insurance was never discussed, and patients’ voluntary health insurance and the reimbursement of the costs of a medical aid were rarely discussed. Both GPs and patients initiated the discussion around patients’ insurance and/or OOP expenses. Table 4 contains examples of discussions between the patient and GP about issues concerning patients’ health insurance and/or OOP expenses.

**Table 3:** Issues concerning patients’ health insurance/OOP expenses that patients and GPs brought up during consultations (n = 475)

Subject (n(%))	Discussed (n(%))	Brought up by: (n(%))		
		Patient	GP	Unknown
Current health insurance (e.g. insurer, insurance type)	0 (0.0)	na	na	na
Current voluntary health insurance (e.g. insurance or not, breadth of coverage)	3 (0.6)	1 (33.3)	2 (66.6)	0 (0.0)
Reimbursement of costs of treatment/diagnostics				
Medical professional for which a referral is needed (e.g. hospital, mental health professional)	12 (2.5)	8 (66.7)	3 (25.0)	1 (8.3)
Medical professional for which no referral is needed (e.g. allied health professional, practice nurse in mental health)	10 (2.1)	3 (30.0)	5 (50.0)	2 (20.0)
Reimbursement of medication	15 (3.2)	8 (53.3)	7 (46.7)	0 (0.0)
Reimbursement of a medical aid	2 (0.4)	2 (100.0)	0 (0.0)	0 (0.0)
Deductibles	10 (2.1)	3 (30.0)	7 (70.0)	0 (0.0)
Co-payments	0 (0.0)	na	na	na
Any of the above	45 (9.5)	22 (48,9)	20 (44.4)	3 (6.7)

**Table 4:** Quotes per insurance/OOP expenses issue that was discussed during consultations

Insurance/OOP expenses issue	Quote
Current voluntary health insurance	P.: “Do you happen to know how this works with the insurance?” GP: “Yes, it depends somewhat on what kind of insurance cover you have. Are you insured for physiotherapy in your supplementary insurance?” (P. 25, woman)
Reimbursement of costs of treatment/diagnostics: referral needed	P.: “They do have a contract with [insurer name] don’t they?” GP: “I believe they both have” P.: “[provider name] did not appear on the list.” (P. 73, woman)
Reimbursement of costs of treatment/diagnostics: no referral needed	P.: “The referral date should be for the first occasion” GP: “And you have already been there yesterday” P.: “Yes, no one told me that. I did not know that you changed… You need to have a referral from your GP first” GP: “If you want it reimbursed. If you say that you wish to visit a psychologist, but that you’ll pay for it yourself, then I don’t need to get involved further.” (P. 52, woman)
Reimbursement of medication	GP: “I am not sure if [medication name] will be reimbursed.” (P. 38, woman)
Reimbursement medical aid	P.: “I needed a referral” GP: “[provider name]” Partner p.: “She had had a wheelchair for several days” P.: “When I fell” GP: “And you needed a referral?” P.: “Yes I needed a referral because otherwise I don’t get it reimbursed by my insurance.” (P. 62, woman)
Deductibles	GP: “I shall print a form for a one-off cholesterol and glucose blood test. And a test for kidney function at the same time too. And this will be, I’m afraid, be part of your cost of care that you pay for yourself, your deductible.” (P. 67, man)

## Discussion

Many patients indicate that they, unexpectedly, have to pay the whole or part of the healthcare bill themselves, while they thought that these costs were covered by their insurance [4]. Having information about costs can prevent patients from unpleasant surprises when they receive their bill for treatment [5]. However, issues concerning patients’ health insurance and/or OOP expenses were discussed in only a minority of the GP consultations. If discussed, patients and GPs talked most often about the three subjects: the reimbursement of treatment or diagnostic tests carried out by a medical professional; the reimbursement of medication; or about whether a treatment cost is part of their deductible and therefore must be paid by themselves. While GPs might be expected to consider the OOP expenses associated with different treatment alternatives, our results indicate that few patients discuss the financial impact of their referral or prescription, with their GP.

Given the way the healthcare system is currently organised, we might expect too much from GPs and patients. In the Netherlands, consultations typically last ten minutes. In this time, GPs are expected to undertake a long list of tasks. They should: diagnose the patient; decide on the course of action to take; oversee and co-ordinate their health needs; provide care themselves if possible; refer patients when needed; and, pay attention to patients’ needs, preferences and concerns. Meanwhile, all of this takes place in a really complex healthcare system with different health plans, breadth of coverage, deductibles and co-payments and, consequently, differences of opinion about what is the best treatment alternative for each patient. Useable and understandable information about the quality and cost of treatment from different providers is still often lacking. Besides, taking OOP expenses into account is not always relevant. Currently, most insurers reimburse the treatment costs from all, or most, providers, and treatments often exceed the mandatory and/or deductible excess, or the deductible may have been satisfied already. In addition, it is often not known in advance if, and how, a patient will be treated and, therefore, what OOP expenses are going to be incurred. Research from the US also stresses that although “many patients wanted to know their total cost-share before embarking on a treatment episode, […] both doctors and patients are insulated from the cost of treatment at the point of care, and both desire more transparency around costs” [3]. It might therefore not be surprising that we found in this study that cost related issues are hardly discussed during consultations, although these costs are important to patients [5]. There are several questions that need to be answered by healthcare providers, insurers and policy makers together. These include: To what degree are GPs currently willing and able to take the insurance and /or OOP expenses of all patients into account when treating or referring them; how can we enable GPs to take these issues into account when deciding on the course of action to take; how can we inform patients about the importance of taking their insurance and/or OOP expenses into account when they require medical care; and how can we stimulate and enable them to do so? These questions could not be answered in this study and require further research.

### Comparisons with the existing literature and the relevance of our study

Our results comply with existing literature. Although patients’ involvement in decision-making has increased somewhat over time [20], literature points out that issues concerning patients’ health insurance and/or OOP expenses are rarely discussed during consultations between the patient and doctor [21, 22]. However, although the incidence of conversations relating to costs is generally low, it varies in the published literature [23]. The incidence depends on a number of factors. For example, these may include: the study method used, for example the incidence in the previous year or whether or not costs were ever mentioned; whether the patient or doctor’s perspective is the starting point of the study; the clinical setting of the study, for example cancer or depression; and, the study population, for example oncology patients or patients not adhering to their treatment due to its cost. We already mentioned that although several papers exist on the subject [23], this study is different because we analysed video recordings of routine GP consultations in the Netherlands. Neither did we focus on a specific patient subgroup. Our results are, therefore, not directly comparable to previous existing studies. OOP expenses have recently increased rapidly in the Netherlands. Therefore, observing real-life, GP consultations adds to the existing literature. It is also relevant internationally because elements of the Dutch healthcare system are shared with other countries. Policymakers as well as healthcare providers and insurers could use our results as a basis for discussing their role in helping patients to take treatment costs into account. Ubel et al. (2016) identified various physician behaviours that lead to missed opportunities to reduce OOP expenses that GP’s could use as input to adjust their behaviour relating to the subject. They could, for instance, consider less expensive treatments when patients are burdened by the expenses of a prescribed treatment [5].

### Strengths, limitations and further research

An important strength of this study is that we used real-life video recorded consultations between patients and their GPs. Furthermore, the GPs and patients were unaware of the fact that the mentioning of patients’ healthcare insurance and/or OOP expenses was our focus of interest. A limitation is that we did not ask GPs and patients whether they took matters around patients’ insurance and/or OOP expenses into account. Consequently, we do not know, ultimately, if they did. Neither do we know whether they feel that this should be part of the consultation. Literature points out, for instance, that people may feel that paying for prescriptions is their own concern, but that the cost of medication, nevertheless, influences decisions regarding their care [21]. Besides, discussing cost issues during consultations is irrelevant in certain situations and we could not assess relevance for each case. Finally, our sample of GPs was not drawn at random and the response rate of the GPs in the second study was low. Nevertheless, our sample matches the population of Dutch GPs with regard to age and gender [30].

Further research is needed, for instance: 1) to study patients’ and GPs’ motives for taking issues concerning patients’ health insurance and/or OOP expenses into account or not – for example investigating whether GPs consider discussing OOP expenses to be their task; 2) to investigate if patients and GPs do take these issues into account even when not talking about it during the consultation; 3) to investigate if there are any causal relationships, and; 4) to investigate if patients’ and GPs’ characteristics, for example their demographics or the patient’s condition, determine whether or not these issues are taken into account.

## Conclusions

While GPs and patients are expected to take OOP expenses into account when deciding on the course of action to take, we found that few patients discuss the financial consequences of their referral or prescription with their GP. However, although having information about costs is important for patients, we might be expecting too much both from GPs and their patients. Healthcare providers, as well as insurers, should think about how they can help patients to take treatment costs into account. On the other hand, patients should be informed about why taking their insurance and/or OOP expenses into account, when they require medical care, is beneficial for them. This study is unique because we analysed video recordings of routine consultations between patients and their GPs in the Netherlands, where elements of the healthcare systems of other countries, such as GPs gatekeeper function and rapidly increasing OOP expenses are combined.

## Abbreviations

GP: general practitioner
OOP expenses: out-of-pocket expenses
